# A decade of investment, HIV prevention research and development funding from 2000 through 2011

**DOI:** 10.1186/1742-4690-9-S1-P101

**Published:** 2012-05-25

**Authors:** K Fisher, E Donaldson, LM Green, T Harmon, P Harrison, R Lande, M Warren

**Affiliations:** 1 Avac - Global Advocacy for Hiv Prevention, New York, USA

## Introduction

Since 2004, the HIV Vaccines and Microbicides Resource Tracking Working Group has employed a comprehensive methodology to track resource trends in R&D for biomedical HIV prevention options, including HIV vaccines, microbicides, PrEP, treatment as prevention, vertical transmission prevention and adult voluntary medical male circumcision.

## Materials and methods

Data were collected on annual disbursements by public, private and philanthropic funders for product development, clinical trials and trial preparation, community education and policy advocacy efforts in order to estimate annual investment in HIV prevention R&D. Investment trends were assessed and compared by year, prevention technology type, type of funder and geographic location.

## Results

Since 2000, there has been significant growth in funding support for HIV prevention research and development. However, in 2011 HIV prevention research began to face increased funding pressures as governments worldwide decreased or flat-lined budgets in many areas of global health and as philanthropic donors worked to revise their investment strategies. Competing funding priorities affected funding for HIV prevention R&D. Still, despite those various funding pressures, the field of HIV prevention research progressed significantly in 2011, with new findings that advanced the field and promising new trials underway.

## Conclusions

Monitoring funding trends for HIV prevention research is particularly important at this time of critical juncture between economic uncertainty and the point at which the scientific community has articulated a much clearer pathway to the end of the HIV epidemic. Monitoring funding provides the fact base for policy advocacy around spending levels and allocations that will sustain investments in the research required to build on the success of recent trials; bring novel HIV prevention candidates into the pipeline; and support the follow-on clinical trials needed to assure the safety, immunogenicity, efficacy and acceptability of new HIV prevention products.

